# Transcatheter Resolution of a Failed Neoconfluence in Pulmonary Atresia With Discontinuous Pulmonary Arteries

**DOI:** 10.1016/j.jaccas.2023.102210

**Published:** 2024-01-04

**Authors:** Jorge Alberto Silva-Estrada, Pavel Martinez-Dominguez, José Luis Colín-Ortiz, Alfredo Bobadilla-Aguirre, Nilda Espinola-Zavaleta

**Affiliations:** a Department of Pediatric Cardiology, National Institute of Pediatrics, México City, México; b Department of Nuclear Cardiology, National Institute of Cardiology Ignacio Chavez, Mexico City, Mexico

**Keywords:** congenital heart defect, cyanotic heart disease, pulmonary atresia

## Abstract

We present a case of a full-term newborn with complex congenital heart defects, including single-ventricle physiology and discontinuous pulmonary arteries. Prompt surgical intervention was performed, which involved pulmonary neoconfluence with autologous pericardium graft and systemic-to-pulmonary shunt placement. However, postoperative complications required stenting to address pulmonary artery stenosis.

## Graphical abstract

**Figure:**
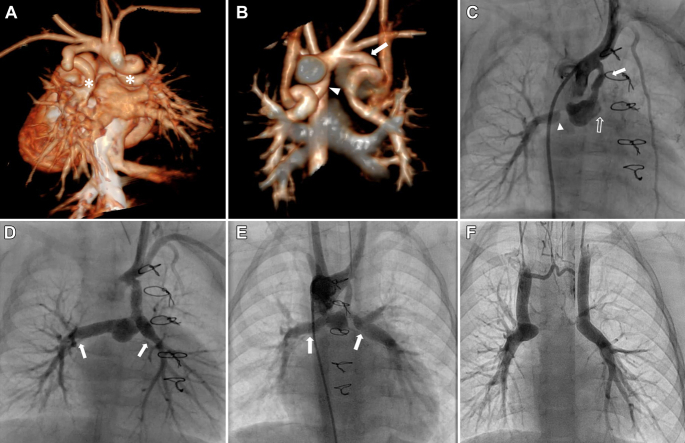

A 2,600-g infant girl was born at 39.6 weeks of gestation to a 29-year-old mother. Her Apgar scores were 3 at 1 minute and 7 at 5 minutes. The Silverman score was 1 because of nasal flaring. The mother had been compliant with adequate prenatal care practices. Notably, a ventricular septal defect was detected during the fifth-month ultrasonography, but no abnormal pulmonary circulation was reported.

Noteworthy vital signs included an oxygen saturation of 86%, along with neonatal central cyanosis. The infant was transferred to the intensive care unit for cephalic helmet placement with FiO_2_ of 30% for 21 hours. Clinical examination revealed a II/VI holosystolic murmur along the left sternal border and single S2. Both preductal and postductal SpO_2_ reached 84%, and the chest x-ray showed slight cardiomegaly, upturned apex, and asymmetrically increased vascular markings. A prostaglandin infusion was initiated, and cardiology consultation was requested.

Transthoracic echocardiography demonstrated abdominal situs inversus, discordant atrioventricular connection with atresia of the left atrioventricular valve, enlargement of the right ventricle, right-handed ventricular topology, hypoplastic left ventricle, single-outlet right ventricle with pulmonary atresia, nonconfluent pulmonary arteries (PA), bilateral superior vena cava without innominate vein, and right aortic arch. Cardiac computed tomography angiography confirmed the anatomy with the additional finding of bilateral ductal origin of the discontinuous branch PA (Figures 1A and 1B).

**Figure 1:**
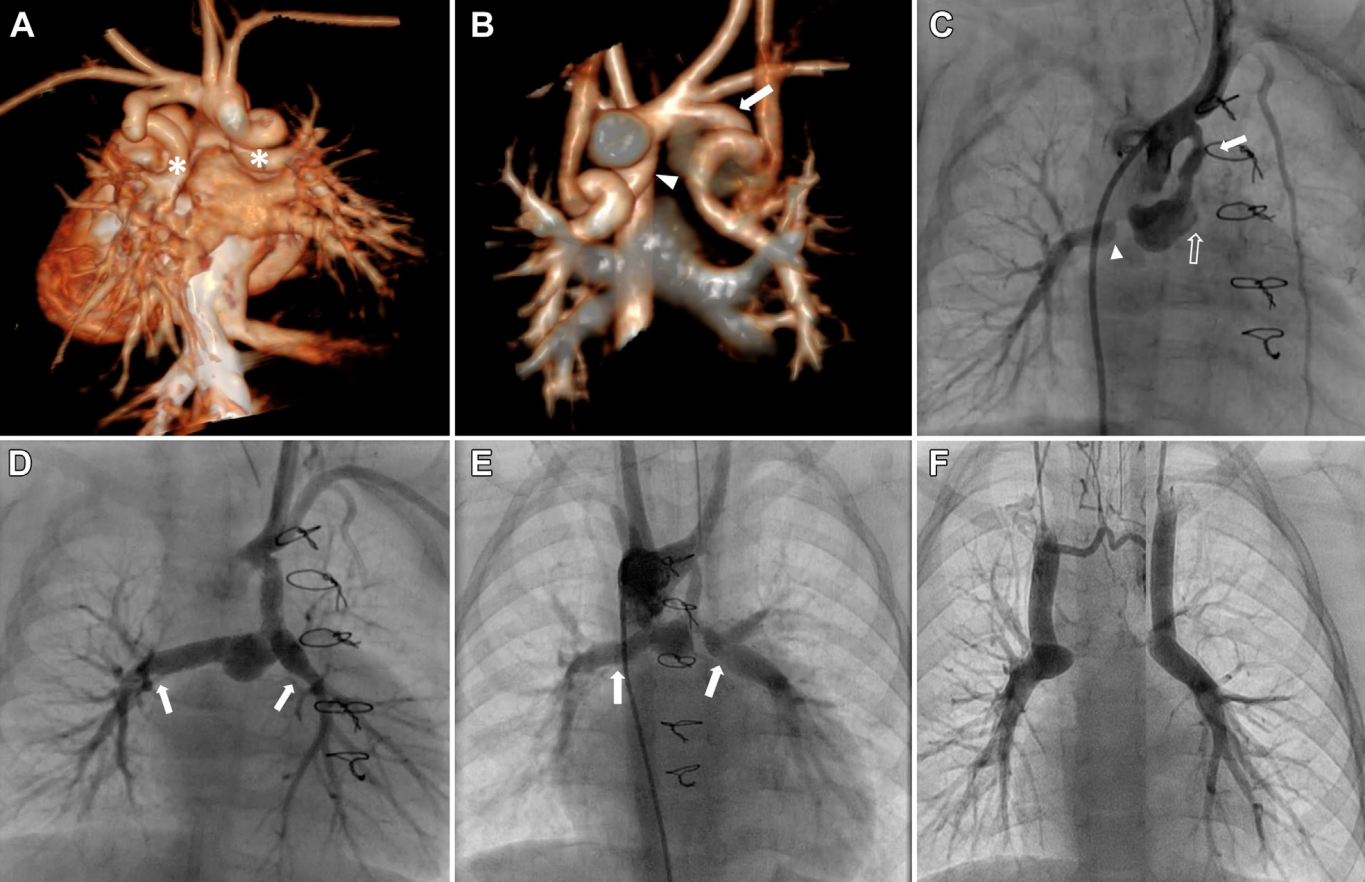
Transcatheter Resolution of a Failed Neoconfluence in Discontinuous Pulmonary Arteries (A, B) Three-dimensional volume-rendered computed tomography angiography shows bilateral ductal origin of both pulmonary arteries (asterisks). Right ductus arteriosus arising from the aortic isthmus (arrowhead) and left ductus arteriosus arising from the left brachiocephalic artery (arrow). (C) Angiography of a right aortic arch shows multiple-site obstruction: junction with right pulmonary artery (arrowhead) and left pulmonary artery (hollow arrow) and proximal portion of systemic-to-pulmonary shunt (white arrow). (D) Angiography after stent placement shows adequate flow in the systemic-to-pulmonary shunt; however, stenosis is evident in the distal right pulmonary artery and distal left pulmonary artery (white arrows). (E) Angiography before Glenn procedure shows the distal portions of both stents (white arrows) at a distance from pulmonary artery branching, as well as significant stenosis of the proximal left pulmonary artery. (F) Patent bilateral Glenn shunts with adequate caliber of distal pulmonary arteries, stents medially alongside the re-stenosed neoconfluence. Abnormal filling of the left upper pulmonary artery is also observed.

The surgical approach involved reconstruction of the discontinuous PA with an autologous pericardium graft. Additionally, a Blalock-Taussig-Thomas shunt (BTTS) was anastomosed to the innominate artery with total resection of ductal tissue. No complications were reported involving the procedure. However, 25 days after the surgery, the patient presented with progressive severe cyanosis. Her vital signs on admission included a heart rate of 138 beats/min, respiratory rate of 50 breaths/min, blood pressure of 90/52 mm Hg, and SpO_2_ of 67%.

In the catheterization laboratory, angiography revealed multiple-site obstruction (Figure 1C, Video 1). Three separate bare-metal, balloon-expandable coronary stents were implanted without complications. Specifically, the first stent was deployed in the right PA, the second in the left PA, and the third in the BTTS stenosis (Figure 1D, Video 2).

Owing to personal circumstances, the patient’s follow-up care was interrupted for 1 year. After re-establishment of follow-up care, a pre-Glenn diagnostic catheterization demonstrated adequate blood flow into appropriate size PA. However, it also showed stenosis of the proximal left PA (Figure 1E). Subsequently, a bilateral bidirectional cavopulmonary shunt procedure was successfully performed, anastomosing both cava distal to the site of the pulmonary stent placement (Figure 1F). The patient is currently awaiting a Fontan repair. She is remarkably asymptomatic, with an SpO_2_ of 75% to 80%.

Bilateral involution of the proximal sixth aspect of the aortic arch results in the absence of the proximal PA.^1^ Discontinuous PA and ductal origin of PA are related conditions that can present simultaneously, as occurred in this case. However, their causes are different.

The management pathway for these cases involves initial palliation through either BTTS or ductal stenting. Although BTTS was traditionally favored, recent reports indicate that ductal stenting is a good alternative that offers enhanced early hemodynamic stability and a shorter length of stay in the hospital. However, it may increase the risk of requiring reintervention.^2^ The Glenn procedure is considered the intermediate step in this palliative series, which concludes with a Fontan repair, allowing direct venous blood flow to the pulmonary circulation. However, questions regarding the optimal timing for centralization repair have come to light. This procedure can be performed at the time of BTTS placement, together with the bidirectional cavopulmonary shunt, or concomitantly with the Fontan repair.^3^

This case highlights the complexity of a single ventricle with discontinuous PA and the importance of carefully assessing the different management options. Further research is essential to provide valuable evidence regarding the optimal timing for centralization surgery and long-term outcomes.

## Funding Support and Author Disclosures

The authors have reported that they have no relationships relevant to the contents of this paper to disclose.

## Appendix

Video 1 Multisite Obstruction Angiography Obstruction of the right aortic arch junction with the right and left pulmonary artery and the proximal portion of the systemic-to-pulmonary shunt.

Video 2 Triple Stent Placement Angiography Angiography shows the first stent placed in the right pulmonary artery, second in the left pulmonary artery, and third in the Blalock-Taussig-Thomas shunt (BTTS). Angiography after stent placement reveals adequate flow in both pulmonary arteries.
